# Alpha-galactosylceramide enhances protective immunity induced by DNA vaccine of the SAG5D gene of *Toxoplasma gondii*

**DOI:** 10.1186/s12879-014-0706-x

**Published:** 2014-12-20

**Authors:** Gang Lu, Aihua Zhou, Min Meng, Lin Wang, Yali Han, Jingjing Guo, Huaiyu Zhou, Hua Cong, Qunli Zhao, Xing-Quan Zhu, Shenyi He

**Affiliations:** Department of Parasitology, Shandong University School of Medicine, Jinan, 250012 Shandong Province China; Department of Pediatrics, Provincial Hospital Affiliated to Shandong University, Shandong University School of Medicine, Jinan, 250021 Shandong Province China; State Key Laboratory of Veterinary Etiological Biology, Key Laboratory of Veterinary Parasitology of Gansu Province, Lanzhou Veterinary Research Institute, Chinese Academy of Agricultural Sciences, Lanzhou, 730046 Gansu Province China

## Abstract

**Background:**

Toxoplasmosis caused by the intracellular parasite *Toxoplasma gondii* (*T. gondii*) is a global epidemic parasitic disease. DNA vaccines play an important role in preventing the spread of toxoplasmosis. SAG family genes encoding particular surface proteins of *T. gondii* are the best candidates of DNA vaccine. As a member of SAG family genes, SAG5 gene has been proved to have better antigenic than SAG1. In addition, alpha-Galactosylceramide (α-GalCer) was used to be an adjuvant in malaria vaccine and received positive results. In this study, the effect of the DNA vaccine enhanced by α-GalCer was evaluated by immunizing BALB/c mice.

**Methods:**

In the present study, SAG5D gene of *T. gondii* was cloned, sequenced, and biologically characterized. BALB/c mice were randomly divided into five groups, including three experimental groups (pEGFP-C1-SAG5D, α-GalCer and α-GalCer/pEGFP-C1-SAG5D) and two control groups (PBS and pEGFP-C1), and were immunized intramuscularly three times. The levels of IgG antibodies and cytokine productions in mouse sera were determined by enzyme-linked immunosorbent assays (ELISA). Two weeks after the last immunization, all mice were challenged intraperitoneally with 1 × 10^4^ tachyzoites of *T. gondii* and the survival time of mice was recorded.

**Results:**

A significant level of increase of IgG response against the soluble tachyzoite antigens (STAg) was detected by ELISA in experimental group. It revealed relatively high level of IFN-γ production by the spleen cells. There were higher productions of interleukin-4 (IL-4) in α-GalCer treated groups compared to control groups. Challenge experiment showed a longer survival period (11 days compared with 5 days in control) in SAG5D DNA vaccinated mice was found after a lethal challenge with *T. gondii* RH strain.

**Conclusions:**

The present study suggested that *T. gondii* SAG5D was a novel and positive DNA vaccine candidate against toxoplasmosis. In addition, the adjuvant (α-GalCer) enhanced the body’s cellular immune response and prolonged the survival time of mice after challenge.

**Electronic supplementary material:**

The online version of this article (doi:10.1186/s12879-014-0706-x) contains supplementary material, which is available to authorized users.

## Background

Toxoplasmosis caused by the intracellular parasite *T. gondii* is a global epidemic parasitic disease, and one in three people worldwide were infected by *T. gondii* in different degrees [1]. In the past decades, the job using DNA vaccines to combat toxoplasmosis had made considerable progress. A series of Toxoplasma-specific genes (SAG, ROP, MIC, GRA, etc.) are widely used to be candidates of DNA vaccines [2]-[7]. SAG family genes encoding particular surface proteins of *T. gondii* are the best candidates, especially SAG1 [8],[9]. SAG1 was considered to be a successful DNA vaccine, because mice achieved excellent immune protection when injected by single SAG1 gene [10],[11] or mult-gene [12],[13] vaccine. Furthermore, SAG2 and SAG3 proteins also have outstanding immunogenicity, and they have been made into a great quantity of encouraging vaccines [14],[15]. SAG5 gene is an important member of SAG family genes and contains five subtypes from SAG5A to SAG5E [16],[17]. SAG5E is a transcribed pseudogene, while SAG5A protein is not expressed in RH strain tachyzoites. The other three SAG5 subtypes can express corresponding proteins in tachyzoites [18]. And the 367 amino acid-long SAG5B and SAG5C are 97.5% identical to each other, while SAG5D amino acid is 50% identical to either of them [19]. As a member of SAG family genes, SAG5 gene may have excellent immunogenicity like SAG1. Accordingly, it is necessary and interesting to construct a SAG5 gene vaccine and examine its immunization.

NKT cells are a group of special T-cell subsets that have T cell receptors and NK cell receptors [20]. Actived NKT cells produce a large number of IL-4 and strongly generate IFN-γ [21]. In immunocompetent hosts, the robust T cell response controls parasite growth via the protective cytokine IFN-γ. α-GalCer is the powerful NKT agonist and is presented by the nonclassical MHC molecule CD1d in both mice and humans [22],[23]. A large number of studies performed in animal models indicated a central role of α-GalCer in stimulation of the netural killer T cells [24],[25]. In previous study, α-GalCer was used to be an adjuvant in some vaccines and received positive results [24]. So it is worth trying to add α-GalCer to DNA vaccines of *T. gondii.*

In our previous study, we analyzed the antigenic characteristics of SAG5B, SAG5C, SAG5D and SAG1 by bioinformatics analysis. SAG5D was selected to be experimental subject because of its positive B-cell epitope index. Now, an eukaryotic expression vector named pEGFP-C1-SAG5D was constructed to determine whether this DNA vaccine can actively elicit immune responses to *T. gondii*. The present work showed that the protection against toxoplasmosis in BALB/c mice caused by SAG5D gene vaccine was obvious. Besides, α-GalCer was used to enhance the immune responses induced with the eukaryotic expression vector.

## Methods

### Mice and parasites

Six- to eight-week-old female BALB/c mice were obtained from Shandong University Laboratory Animal Center. All mice were bred in groups of eight per cage under specific-pathogen-free conditions and had free access to diet and tap water. All of the animal experiments were approved by the Ethics Committee on Animal Experiments of the Medical School of Shandong University.

The RH strain of *T. gondii* was maintained in our laboratory by passage of tachyzoites in BALB/c mice. The *T. gondii* tachyzoites used in experiment were harvested from the peritoneal fluid of mice 72 hr after infection. Most of the tachyzoites were used to create soluble tachyzoite antigens (STAg) after washed by centrifugation and resuspended in sterile PBS, while the rest were used to extract the total RNA of RH strain. To ensure the freshness of tachyzoites used for challenge, tachyzoites were extrated and purified from mice 1 hr before injection.

### Plasmid construction and preparation

The entire SAG5D open reading frame (ORF) was amplified by PCR from the cDNA of *T. gondii* (RH strain) tachyzoites with primer (5′- cggGGTACCATGGTGCGACGGTCTTC -3′)(forward) and primer (5′-cgcGGATCCTCAATATGTGCCAAGA -3′)(reverse). Both of the two primers contain Kpn I and BamH I restriction sites. PCR amplification was performed using the following conditions: 1 cycle of 95°C for 5 min then 30 cycles of 95°C for 30 sec, 59°C for 30 sec, and 72°C for 1 min. Final primer extension was extended to 10 min at 72°C. PCR product was analyzed by electrophoresis on 1.0% agarose gel.

The PCR products of SAG5D gene was cloned into a pEASY-T1 vector (TransGen Biotech, China) to generate a recombinant cloning plasmid. Sequence determination was finished by a professional company (Sheng Gong, Shanghai). After sequenced, SAG5D was subcloned into a eukaryotic expression plasmid pEGFP-C1 (Novagen, USA) to produce pEGFP-C1-SAG5D. Finally, the new recombinant plasmids were transfected into HEK 293-T cells with Lipofectamine™ 2000 reagent (Invitrogen, USA).

### Plasmid extraction and purification

Large-scale plasmids were extracted with the Endotoxin-Free Mega kit according to the manufacturer’s instructions (Qiagen, Hilden, Germany), and the concentrations were determined by A260/A280 measurement. The ratios of the best densities at 260 and 280 nm (OD260 and OD280, respectively) was 1.8, indicating few protein contamination. Plasmid DNA was diluted into 1 mg/ml with sterile endotoxin-free PBS and stored at −20°C before use.

### Expression of SAG5D in HEK 293-T cells

HEK 293-T cells, an embryonic kidney cell line, were grown at 37°C in a humidified 5% CO_2_ atmosphere in 6-well plates (Costar, USA) in Dulbecco’s Modified Eagle Medium (DMEM) containing 100 mg/ml streptomycin and 100 IU/ml penicillin and 10% fetal Bovine serum. When the density of HEK 293-T cells reached 80%-90%, the constructed eukaryotic expression plasmids (pEGFP-C1-SAG5D) and the empty vectors (pEGFP-C1) were transfected into cells by the Lipofectamine 2000 regent (Invitrogen, USA) on the basis of the manufacturer’s guidance. Plasmids (pEGFP-C1-SAG5D and pEGFP-C1) were all mixed with lipofectamine 2000 reagent at a concentration of 10 μg/ml in DMEM without Fetal Bovine Serum (FBS) and antibiotics. The mixed solutions were incubated at room temperature for 20 min before added into HEK 293-T cells drop by drop. The cells were incubated with the transfection mixture for 6 h at 37°C in a humidified 5% CO_2_ atmosphere. Finally, fresh cell culture fluid was added and the 6-well plates were returned to the cell incubator for 48 hr incubation.

The cells from different groups (blank, pEGFP-C1 and pEGFP-C1-SAG5D) were respectively observed under fluorescence microscope under blue laser after incubation. HEK 293-T cells were collected and the expression of target gene was evaluated by Western blotting analysis.

### Western blot analysis

Western blot analysis of HEK 293-T cells transfected with pEGFP-C1-SAG5D was performed as below: The cells were treated using RIPA Lyses Buffer (50 mM Tris pH 7.4, 150 mM NaCl, 1% Triton X-100, 1% Sodium deoxycholate, 0.1% SDS) containing 1 mM protease inhibitor PMSF (phenylmethanesulfonyl fluoride) and centrifuged at 13,000 × g for 10 min. Then supernatant was extracted and resuspended in 50 μl of SDS-PAGE sample buffer, and boiled for 5 min and 20 μl was loaded onto a 12% polyacrylamide gel. Proteins were transferred onto PVDF (polyvinylidenefluoride) membrane via electrophoresis, carried at 60 V for 4 hr, using Bio-Rad transfer system (Bio-Rad, Hercules, CA). The membrane was saturated for 2 hr with sealing fluid at room temperature and probed with anti-*T. gondii* polyclonal antibody (Goat) diluted 1:10000 in saturation buffer. The membrane was incubated for 2 hr with a HRP (horseradish peroxides)-labeled rabbit anti-goat IgG antibody (Sigma, USA) diluted 1:20,000 in saturation buffer, and signals were detected with super sensitive signal ECL (Enhanced Chemiluminescence) system.

### Expression of gene in vivo

To examine the level of expression in vivo, total RNA was extracted from spleens of DNA-vaccinated mice 2 weeks after the last immunization and analyzed by RT-PCR with SAG5D-specific primers. RT-PCR products were then analyzed by agarose gel electrophoresis.

### DNA immunization and experimental design

Five groups (16 mice per group) mice were vaccinated twice at 2-week intervals with PBS (100 μl/each), empty vector (100 μg/each), α-GalCer (2 μg/mouse) [24], pEGFP-C1-SAG5D (100 μg/each), or pEGFP-C1-SAG5D (100 μg/each) with α-GalCer (2 μg/mouse). Moreover, α-GalCer was only injected at the last time while others were injected intramuscularly three times. The immunization schedules of BALB/c mice were shown in Table 1. Blood was collected to assess serum IgG levels at 2, 4 and 6 weeks after immunization. Two weeks after the final immunization, mice were challenged intraperitoneally with 1 × 10^4^ tachyzoites of *T. gondii* RH strain to determine the survival time.

**Table 1:** **The immunization process of BALB/c mice**

Group	Immunization time ^a^		
	First time	Second time	Third time
PBS	100 μl	100 μl	100 μl
pEGFP-C1	100 μg	100 μg	100 μg
α-GalCer	100 μl PBS	100 μl PBS	2 μg α-GalCer^b^
pEGFP-C1-SAG5D	100 μg	100 μg	100 μg
α-GalCer/pEGFP-C1-SAG5D	100 μg	100 μg	100 μg
		pEGFP-C1-SAG5D	pEGFP-C1-SAG5D/2 μg α-GalCer

^a^All mice were injected three times at two week interval.

^b^2 μg α-GalCer was diluted with 100 μl PBS before use.

### Assays of *T. gondii*-specific IgG and IgG subclass titers

*T. gondii*-specific serum antibody levels were determined by enzyme-linked immunosorbent assay (ELISA) according to previously introduced. Briefly, the 96-well plates (Costar, USA) were coated with STAg (10 μg/well) and incubated at 4°C overnight. Plates were washed three times with special ELISA lotion and blocked with PBS containing 1% Bovine Serum Albumin (BSA) for 2 hr at room temperature. The plates were incubated with the mouse sera diluted by PBS for 1 hr at 37°C. After washing, plates were incubated with horseradish peroxidase (HRP)-conjugated anti-mouse IgG (diluted 1:4,000 in PBS–1% BSA), IgG1 (1:2,000), and IgG2a (1:2,000) for 1 hr at 37°C. After washing with lotion, orthophenylene diamine (Sigma, USA) and 0.15% H_2_O_2_ were added. Plates then were incubated in the dark for 30 min at 37°C, and the reaction was stopped by adding 2 M H_2_SO_4_. The OD was measured at 490 nm using an ELISA reader (ELX800, USA). All samples were run for four times.

### Cytokine quantification

Two weeks after the last immunization, four mice per group were euthanized, and their spleens were isolated in a sterile condition. The spleen cells were cultured in 96-well plates at 37°C in 5% CO_2_. Cell-free supernatants were harvested and assayed for IL-4 activity at 24 hr, for interleukin-10 (IL-10) activity at 72 hr, for gamma interferon (IFN-γ) activity at 96 hr. The IL-4, IL-10, and IFN-γ concentrations were evaluated with a commercial ELISA kit on the basis of the manufacturer’s instructions (R&D Systems, USA).

### Statistical analysis

The software used was SPSS 17.0 for Windows. Antibody production and cytokine levels among the diverse groups were determined using a one-way analysis of variance. The statistical evaluation of the differences in survival rates was checked by Kaplan-Meier test. When a significant difference (P = 0.05) was observed among treatments, Tukey’s studentized range test was used for post-test comparisons. The difference was considered statistically significant if P < 0.05.

## Results

### Identification of the recombinant plasmid

**Figure 1:**
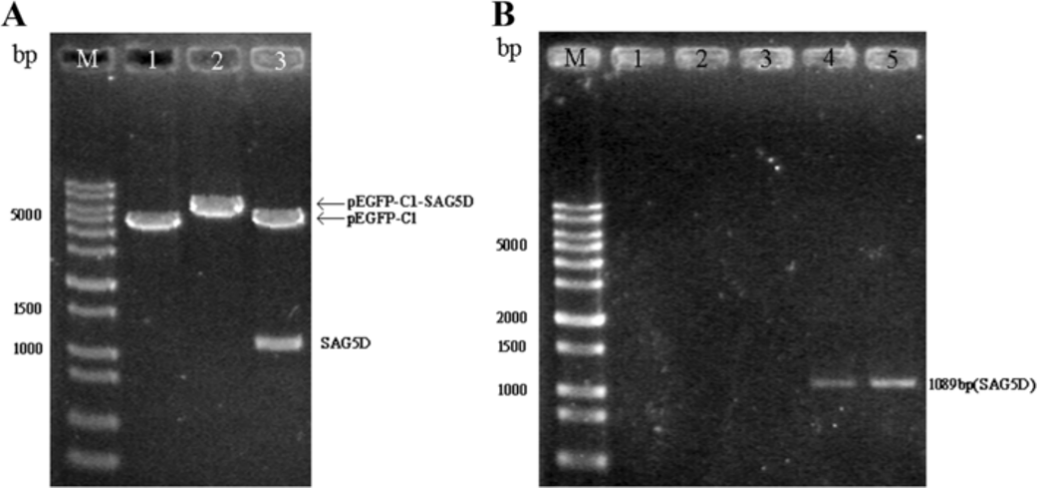
**Identifications of SAG5D gene. (A)** Constructed plasmid maps and identification of the recombinant plasmids with restriction enzyme digestion. DNA Mark (lane M), pEGFP-C1 digested with BamH I (lane 1), pEGFP-C1-SAG5D digested with BamH I (lane 2), pEGFP-C1-SAG5D digested with Kpn I and BamH I (lane 3). **(B)** In vivo expression analysis of the constructs in mice by RT-PCR. DNA Mark (lane M), PBS (lane 1), α-GalCer (lane 2), pEGFP-C1 (lane 3), pEGFP-C1-SAG5D (lane 4), pEGFP-C1-SAG5D/α-GalCer (lane 5).

SAG5D gene was cloned into the eukaryotic expression vector pEGFP-C1 with suitable restriction enzymes to generate a new recombinant plasmid. In order to ensure the accurateness of plasmid, it was tested by PCR and restriction enzyme analysis. The result of restriction enzyme digestion of plasmid was shown in Figure 1A.

### Gene expression *in vitro* and *in vivo*

**Figure 2:**
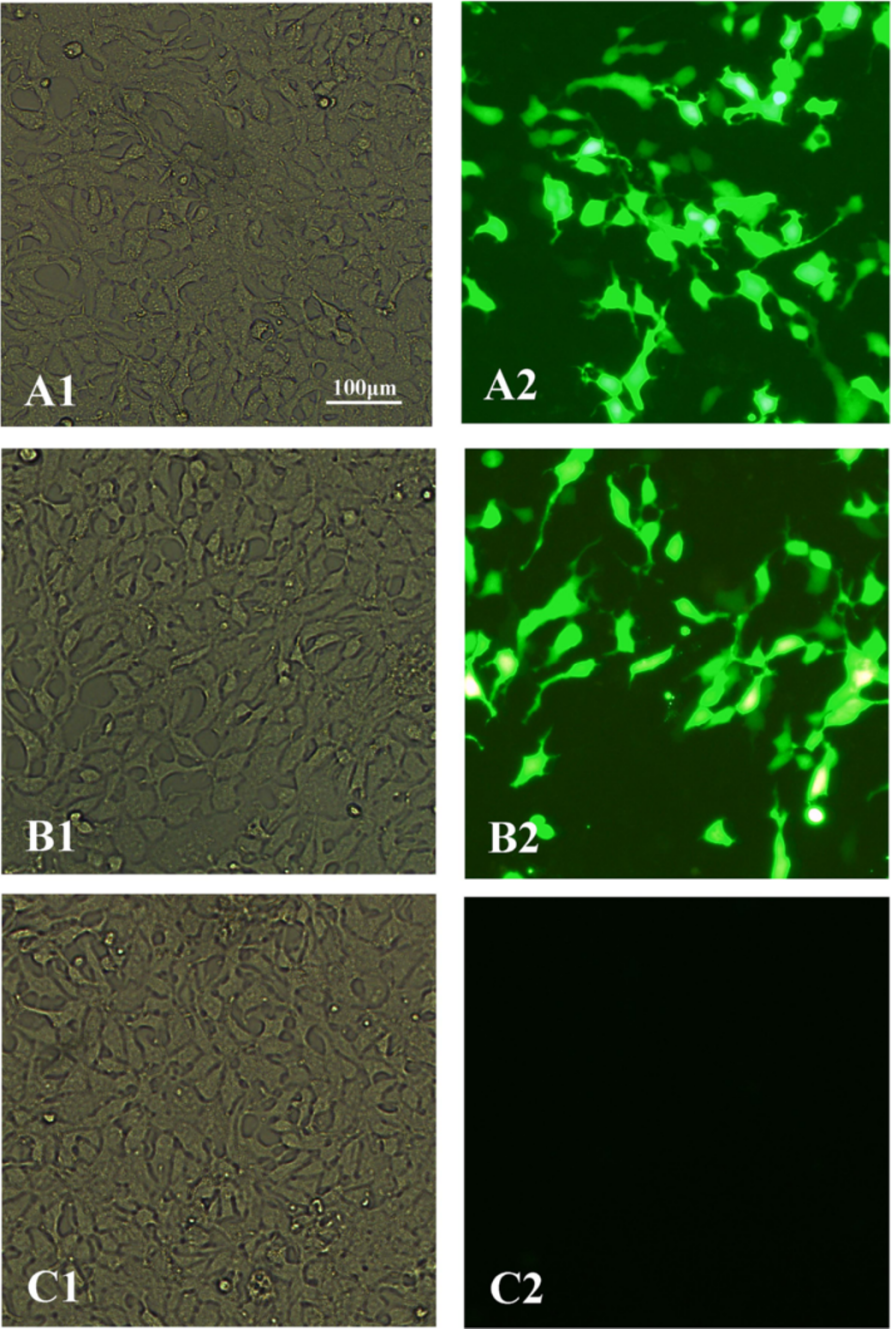
**Direct immunofluorescence detection of the fusion protein in transfected HEK 293-T cells. (A1)** Cells transfected with pEGFP-C1-SAG5D detected under white light; **(A2)** Cells transfected with pEGFP-C1-SAG5D detected under blue light; **(B1)** Cells transfected with pEGFP-C1 detected under white light; **(B2)** Cells transfected with pEGFP-C1 detected under blue light; **(C1)** Untransfected cells under white light; **(C2)** Untransfected cells under blue light.

HEK 293-T cells were successfully transfected with pEGFP-C1-SAG5D and pEGFP-C1 for 48 hr. In pEGFP-C1-SAG5D and pEGFP-C1 transfected cells, the proteins were excited green fluorescence under blue laser using fluorescence microscope (Figure 2A and B), whereas no fluorescence was observed in blank cells (Figure 2C).

**Figure 3:**
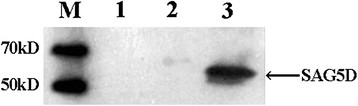
**In vitro expression analysis of the constructs in HEK 293-T cells by Western blotting.** Protein marker (lane M), untransfected cells (lane 1), cells transfected with pEGFP-C1 (lane 2), cells transfected with pEGFP-C1-SAG5D (lane 3).

The inserted gene expression was detected by Western blotting. As shown in Figure 3, the expression of SAG5D gene (about 60 kDa, compound protein) was detected in pEGFP-C1-SAG5D transfected cells, while no band was found in empty vector transfected and blank cells.

Furthermore, the expression of gene SAG5D in mice was detected with RT-PCR. Electrophoresis of RT-PCR products from two groups (pEGFP-C1-SAG5D and α-GalCer/pEGFP-C1-SAG5D) showed the expected fragment of the SAG5D gene (1089 bp) (Figure 1B). Whereas there was no band in the other three groups (PBS, α-GalCer and pEGFP-C1).

### Antibody responses in immunized BALB/c mice

**Figure 4:**
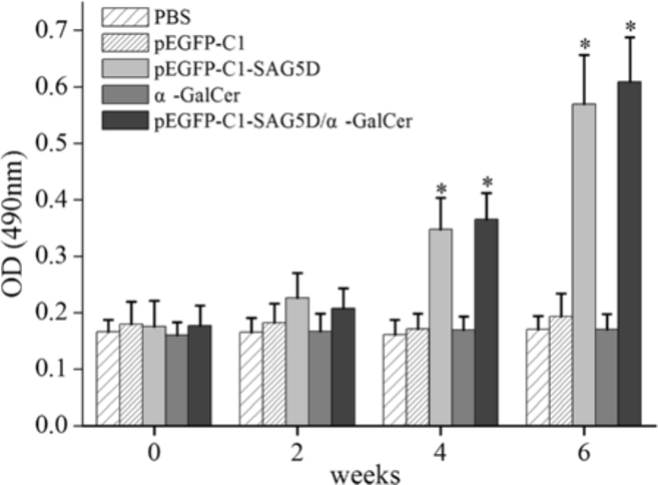
**Measurement of specific IgG antibodies in sera of immunized mice.** Sera were collected two days prior to each immunization and determined by ELISA. Results are shown as means of OD 490 ± SD and statistical differences (P < 0.05) are indicated by * compared with PBS, α-GalCer or pEGFP-C1.

The levels of IgG antibodies induced by α-GalCer and plasmids in mice were detected by ELISA at weeks 0, 2, 4 and 6. As shown in Figure 4, significantly high levels of IgG antibodies were detected in the sera of mice vaccinated with pEGFP-C1-SAG5D or α-GalCer/pEGFP-C1-SAG5D, especially after the third immunization. Furthermore, mice injected with PBS, α-GalCer or pEGFP-C1 alone also generated IgG antibodies. But the levels of IgG antibodies were significantly lower than those of mice immunized with pEGFP-C1-SAG5D or α-GalCer/pEGFP-C1-SAG5D (P < 0.05). There was no statistical difference between PBS and α-GalCer, PBS and pEGFP-C1 or α-GalCer and pEGFP-C1 (P > 0.05). The level of IgG antibodies from mice injected with α-GalCer/pEGFP-C1-SAG5D was higher than those of pEGFP-C1-SAG5D, but there was no statistical difference between them (P > 0.05). All the results strongly illustrated that a new plasmid encoding *T. gondii* SAG5D protein induced a strong IgG antibody response in mice.

**Figure 5:**
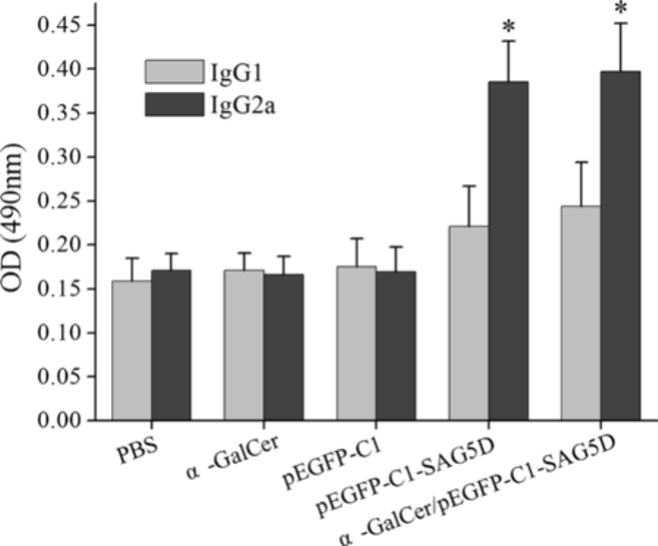
**Detection of IgGl and IgG2a levels in the vaccinated mouse sera by ELISA.** Immune sera were collected at 2 weeks after the last immunization and determined by ELISA. Results are shown as means of OD 490 ± SD and statistical differences (P < 0.05) are indicated by * as compared to control groups.

The levels of IgG subclass (IgG1 and IgG2a) in all groups at the second week after the final injection were analyzed to determine whether a Th1 or Th2 response was stimulated and the outcomes were shown in Figure 5. An apparent predominance of IgG2a over IgG1 was observed in pEGFP-C1-SAG5D or α-GalCer/pEGFP-C1-SAG5D vaccine immunized mice, which indicated a shift toward the Th1 type response. In addition, higher level of IgG2a in mice vaccinated with α-GalCer/pEGFP-C1-SAG5D was detected than that injected by PBS, α-GalCer or pEGFP-C1 (P < 0.05). Moreover, there was no statistical difference in IgG2a levels between the groups immunized with pEGFP-C1-SAG5D and α-GalCer/pEGFP-C1-SAG5D (P > 0.05). The result showed that pEGFP-C1-SAG5D immunized mice produced specific antibodies against *T. gondii* SAG5D and mainly generated a Th1 immune response.

### Cytokine production

To determine whether pEGFP-C1-SAG5D or α-GalCer/pEGFP-C1-SAG5D immunization augmented the Th1 or Th2 cytokine response, purified pEGFP-C1-SAG5D- and α-GalCer/pEGFP-C1-SAG5D-treated culture supernatants of splenocytes were obtained from immunized mice 2 weeks after the last immunization. As shown in Table 2, the levels of IFN-γ in mice immunized with α-GalCer, pEGFP-C1-SAG5D, or α-GalCer/pEGFP-C1-SAG5D vaccines were significantly higher than those of mice immunized with PBS or empty vector (P < 0.05). Furthermore, the level of IFN-γ in mice injected by α-GalCer/pEGFP-C1-SAG5D was significantly higher than that of mice injected by α-GalCer or pEGFP-C1-SAG5D (P < 0.05). There was no statistical difference in IFN-γ levels between the groups immunized by PBS and empty vector. The levels of IL-4 in mice immunized with α-GalCer or α-GalCer/pEGFP-C1-SAG5D were two times as high as those of mice immunized with pEGFP-C1-SAG5D, PBS, or empty vector (P < 0.05). In addition, the low levels of IL-10 shown in the experimental and control groups suggested there was no statistically significant differences among the groups (P > 0.05).

**Table 2:** **Cytokine production by splenocyte** ^**a**^ **cultures from immunized BALB/c mice**

Group	Cytokine production (pg/mL) ^b^		
	IFN-γ	IL-4	IL-10
PBS	52.07 ± 8.23	36.36 ± 5.25	36 ± 4.98
pEGFP-C1	53.15 ± 8.71	39.24 ± 6.43	37.4 ± 6.33
α-GalCer	343.6 ± 50.88^*^	75.46 ± 5.7^*^	37.56 ± 3.66
pEGFP-C1-SAG5D	573.43 ± 60.2^*^	36.65 ± 3.52	40.77 ± 5.66
α-GalCer/pEGFP-C1-SAG5D	781.36 ± 57.57^*#^	76.73 ± 6.08^*^	41.33 ± 5.72

^a^Splenocytes from 4 mice per group two weeks after the final immunization. ^b^Values for IFN-γ at 96 hr, IL-4 at 24 hr, IL-10 at 72 hr are expressed as mean ± SD. ^*^Compared with PBS or pEGFP-C1 group, P < 0.05; ^#^compared with pEGFP-C1-SAG5D or α-GalCer, P < 0.05.

### Protection of recombinant plasmid vaccine against *T. gondii*in mice

**Figure 6:**
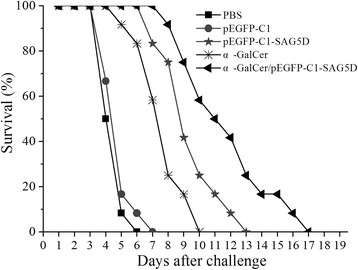
**Survival time.** Survival curves of mice immunized with PBS, pEGFP-C1, α-GalCer, pEGFP-C1-SAG5D or pEGFP-C1-SAG5D/α-GalCer after challenge with 1 × 10^4^ tachyzoites of RH strain 2 weeks after the final immunization (12 mice per group). Survival was monitored daily for 18 days after challenge.

To evaluate the level of immunoprotection induced by the DNA vaccine, all of the mice were challenged intraperitoneally with a lethal dose of *T. gondii* RH strain (1 × 10^4^ tachyzoites). The number of days of survival was noted daily until all of the mice showed signs of illness and were killed (Figure 6). Mice immunized with α-GalCer/pEGFP-C1-SAG5D or pEGFP-C1-SAG5D had dramatically higher survival times than the control mice vaccinated with PBS or empty vector (P < 0.05). Although mice vaccinated with α-GalCer/pEGFP-C1-SAG5D showed a longer survival time than those vaccinated with pEGFP-C1-SAG5D, no statistical difference was observed between them (P > 0.05). Besides, the survival time of mice immunized with α-GalCer was longer than those immunized by PBS or empty vector. But difference between the two groups was not statistically significant for any of them (P > 0.05).

## Discussion

In our previous study, we used bioinformatics approaches to identify antigenic epitopes on SAG5D protease. There was better B-cell epitope index in SAG5D protein than that in SAG1 protein (data not shown). It was theoretically proved that SAG5D protein was a positive antigen like SAG1 protein.

The present study shows that the recombinant plasmid elicited a strong humoral and cellular immune response against *T. gondii* in mice. Levels of specific IgG antibodies, IFN-γ, IL-4, and IL-10 were detected in experimental groups and control groups. Significantly higher levels of IgG2a and IFN-γ were observed in mice injected by pEGFP-C1-SAG5D or α-GalCer/pEGFP-C1-SAG5D compared with control groups. Furthermore, immunization with the gene plasmid pEGFP-C1-SAG5D or α-GalCer/pEGFP-C1-SAG5D induced a great tendency toward Th1-type immune responses. Th1 cytokines can kill intracellular parasites while Th2 cytokines play an important role in worsening infection [26]. In previous studies, IFN-γ promotes Thl cells maturation, while IL-4 promotes Th2 cell maturation [27]. IFN-γ is the key mediator of resistance to *T. gondii* and elicites multiple complicated intracellular mechanisms to kill the parasite and inhibit its replication [28],[29]. Furthermore, IFN-γ plays a key role in protecting hosts from parasites during all phases of toxoplasmosis [30],[31].

NKT cell activation has emerged as a promising strategy for the immunotherapy of cancer, and great effort is committed to developing NKT cell agonists into safe and powerful vaccine adjuvants [32]. The efficacy of lipid antigens, such as the prototypic NKT cell agonist α-galactosylceramide (α-GalCer) [33], is currently being evaluated clinical trials. Furthermore, α-GalCer playing a role of adjuvant in malaria vaccine was studied and achieved some positive results [24].

Here we tested the potential of a-GalCer as an adjuvant during *T. gondii* infection. NKT cells can respond within minutes to specific glycolipids such as α-GalCer [32],[34],[35] and have the capacity to produce high quantities of both Th1 and Th2 cytokines. The main immune components affected by α-GalCer administration are specific CD8+ and CD4+ T cells that secrete IFN-γ [24]. IFN-γ is important in mediating the adjuvant effect of α-GalCer. Studies by a number of different investigators clearly showed that IFN-γ is secreted by both NKT and NK cells after α-GalCer treatment [36]-[38]. It is possible that IFN-γ secreted by NKT and/or NK cells acts on antigen-presenting cells, by up-regulating the major histocompatibility complex class 1 (MHC-1) processing machinery. Successful DNA vaccination is in theory well suited to stimulate effector mechanisms depending on antigen presentation along with MHC-1, which stimulate CD8^+^ cytotoxic T cells [39],[40].

The levels of the humoral response and the Th2 response are nearly unaltered by this treatment. Furthermore, IFN-γ may enhance the acquired cell-mediated immune response by directly acting on antigen-specific CD8^+^ T cells [24],[41],[42]. Nevertheless, the exact role of IFN-γ and other critical molecules requires further investigation. In addition, some study results have indicated that α-GalCer treatment enhances the immune response to viral infection, promoting long-term immunity without disrupting the original immune response. Moreover, α-GalCer or an α-GalCer analog could also be used therapeutically to control latent infection when the immune system is impaired [24],[43]. All testify that α-GalCer is a perfect activator of the immune response in vivo. Recent studies have evaluated a-GalCer as a potential adjuvant due to its ability to induce the activation of immune cells. In contrast to currently used vaccine adjuvants, a-GalCer may be a viable adjuvant to boost cellular responses as it has been shown to enhance cellular immune responses to toxoplasma antigens when used as adjuvant of SAG5D DNA vaccine.

The survival times of all of the mice in the five groups after intraperitoneal challenge with 1 × 10^4^ tachyzoites of the RH strain were recorded. Mice immunized with α-GalCer had more IFN-γ and longer survival times than mice in PBS and pEGFP-C1 groups. The results described above indicated that α-GalCer did play a positive role in preventing mice from being infected with tachyzoite. The survival times of mice injected by pEGFP-C1-SAG5D were similar to that of mice immunized with pSAG1 [13]. All of the mice showed signs of illness and were killed on the 17^th^ after challenge.

## Conclusions

The present study suggested that *T. gondii* SAG5D was a novel and positive DNA vaccine candidate against toxoplasmosis. Toxoplasma SAG5D nucleic acid vaccine (pEGFP-C1-SAG5D) is simply prepared and easy to be produced. Furthermore, it has a strong immunogenicity in mice after injection. It can induce the body to properly produce strong cellular and humoral immune response, which play an important role in immune protection. In order to improve DNA vaccine protection, a kind of suitable adjuvant (a-GalCer) was added. It enhanced the body’s cellular immune response and prolonged the survival time of mice after challenge.

## Authors’ original submitted files for images

Below are the links to the authors’ original submitted files for images. Authors’ original file for figure 1 Authors’ original file for figure 2 Authors’ original file for figure 3 Authors’ original file for figure 4 Authors’ original file for figure 5 Authors’ original file for figure 6
