# Supplementary figures and images for: Alpha-galactosylceramide enhances protective immunity induced by DNA vaccine of the SAG5D gene of Toxoplasma gondii
Source: BMC Infect Dis. 2014 Dec 20;14:3862. doi: 10.1186/s12879-014-0706-x (PMC4312432; doi:10.1186/s12879-014-0706-x)

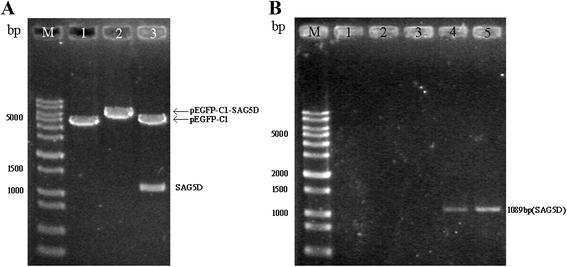

Supplement: Supplementary file 1 — Authors’ original file for figure 1 [file 12879_2014_706_MOESM1_ESM.gif]

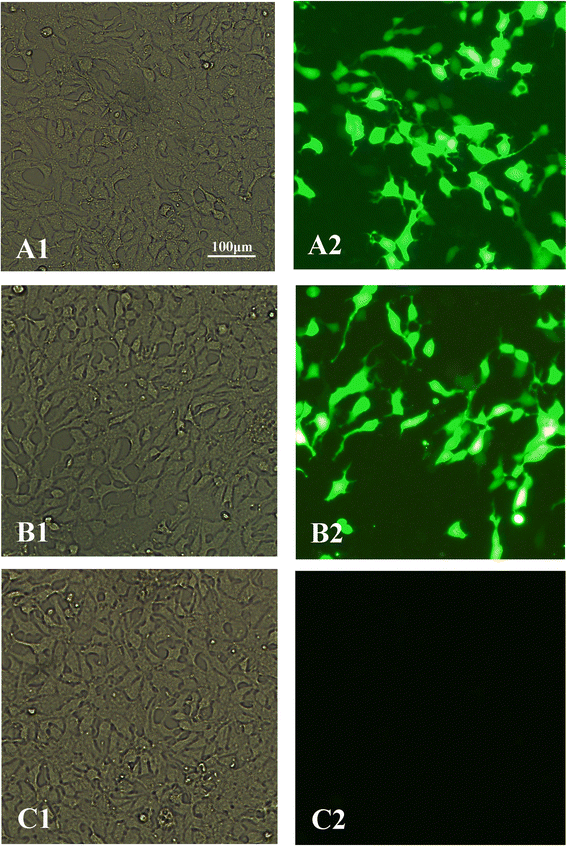

Supplement: Supplementary file 2 — Authors’ original file for figure 2 [file 12879_2014_706_MOESM2_ESM.gif]

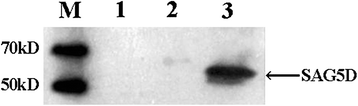

Supplement: Supplementary file 3 — Authors’ original file for figure 3 [file 12879_2014_706_MOESM3_ESM.gif]

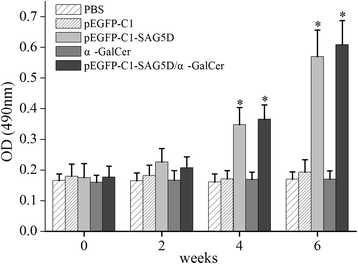

Supplement: Supplementary file 4 — Authors’ original file for figure 4 [file 12879_2014_706_MOESM4_ESM.gif]

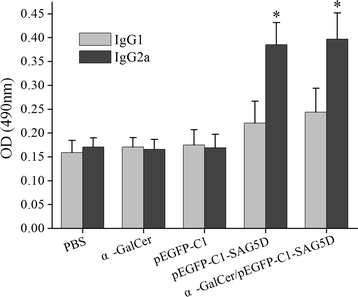

Supplement: Supplementary file 5 — Authors’ original file for figure 5 [file 12879_2014_706_MOESM5_ESM.gif]

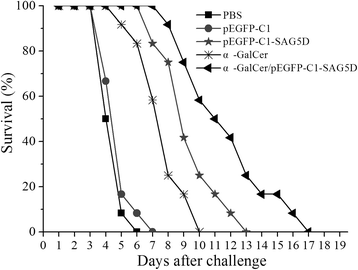

Supplement: Supplementary file 6 — Authors’ original file for figure 6 [file 12879_2014_706_MOESM6_ESM.gif]
